# Low-molecular-weight fucoidan and high-stability fucoxanthin from brown seaweed exert prebiotics and anti-inflammatory activities in Caco-2 cells

**DOI:** 10.3402/fnr.v60.32033

**Published:** 2016-08-02

**Authors:** Pai-An Hwang, Nam Nhut Phan, Wen-Jung Lu, Bui Thi Ngoc Hieu, Yen-Chang Lin

**Affiliations:** 1 Department of Bioscience and Biotechnology, National Taiwan Ocean University, Keelung, Taiwan; 2 Graduate Institute of Biotechnology, Chinese Culture University, Taipei, Taiwan; 3 Faculty of Applied Sciences, Ton Duc Thang University, Hồ Chí Minh, Vietnam; 4 Department of Food Science, National Taiwan Ocean University, Keelung, Taiwan

**Keywords:** low-molecular-weight fucoidan, fucoxanthin, anti-inflammatory, prebiotics

## Abstract

**Background:**

The aim of this study is to investigate the anti-inflammatory effects of low-molecular-weight fucoidan (LMF) and high-stability fucoxanthin (HS-Fucox) in a lipopolysaccharide-induced inflammatory Caco-2 cell line co-culture with *B. lactis*.

**Methods:**

We used various methods such as transepithelial resistance (TER) assay, cytokine secretion assay, and tight junction protein mRNA expression assay to examine LMF and HS-Fucox anti-inflammatory properties.

**Results:**

LMF and HS-Fucox activated probiotic growth and reduced the inflammation of the intestinal epithelial cells. Moreover, the combination of LMFHS-Fucox dramatically enhanced the intestinal epithelial barrier and immune function against the lipopolysaccharide effect by inhibiting IL-1β and TNF-α and promoting IL-10 and IFN-γ.

**Conclusion:**

These findings suggested that LMF and HS-Fucox, alone or in combination, could be the potential natural compounds to enhance the immune system and have an anti-inflammatory effect on the intestinal cells.

The gastrointestinal tract is constantly exposed to and heavily populated with potentially pathogenic microorganisms. Thus, the immune system maintains a strong presence at the mucosal boundary, and the digestive tube is heavily laden with lymphocytes, macrophages, and other cells that participate in immune responses. Intestinal epithelial cells are the first line of defense, which separate the host's internal milieu from the external environment and play an important role in the maintenance of immune homeostasis (1). Therefore, the intestinal epithelial cells preserve the barrier function by producing defenses, such as cytokines, and maintaining cell tight junctions. Intestinal epithelial cells can produce several chemokines and pro-inflammatory cytokines in response to pathogens to induce the migration of granulocytes, lymphocytes, and dendritic cells, resulting in the induction of host immunity (2). Intestinal epithelial tight junctions act as an intercellular gate, serving as a physical barrier against the paracellular permeation of luminal antigens. The tight junctions consist of four integral membrane proteins: occludins, claudins, tricellulin, and the junctional adhesion molecule, which completely encircle the apex of the cell and make contact with the tight junctions of adjacent cells, forming a continuous paracellular seal (3). It is generally believed that a disease-related increase in intestinal permeability is caused by defects in the tight junctions’ structure and functions, and the subsequent development of intestinal inflammation (4).

*Bifidobacterium* spp. are a natural inhabitant of the gastrointestinal tract and they play the role of health-promoter or probiotic bacteria. The adhesion of bifidobacterial strains to the human intestinal epithelium has been proposed as one of the main reasons for immune modulation and the stimulation of the healing of damaged intestinal cells (5). Recent reports indicated that the adhesion of bifidobacterial strains to human intestinal cells is independent of the receptors for bacterial adhesions on mucus (6, 7). Furthermore, the adhesive properties of the bifidobacterial strains are a key determinant for the cytokine production by enterocytes, and probably the initiating event in probiotic immunomodulatory activity, as it occurs prior to the encounter with the immune system cells (8). Among them, *Bifidobacterium lactis* is a probiotic strain that is consumed worldwide, which can reduce the severity of pathogen infection and enhance immunity in the elderly (9) and protect enterocytes from an acute inflammatory response induced by enteropathogen (10).

Kennedy and Sandin first reported that polysaccharides can significantly enhance cell adhesion and hydrophobicity as prebiotics (11). Brown seaweeds are an important source of bioactive polysaccharides and other compounds. Fucoidan is a class of sulfated and fucosylated polysaccharides found in brown seaweeds (12), and a recent study demonstrates that low-molecular-weight fucoidan shows more potent bioactivities than high-molecular-weight fucoidan (13). Our previous studies also have demonstrated that low-molecular-weight fucoidan has good bioactivities in vitro and in vivo (14–17). The pharmacological activity of fucoidan in intestinal inflammation has been provided in animal models (18–21), but the pharmacological activity of low-molecular-weight fucoidan still unknown. Fucoxanthin, a carotenoid present in the chloroplasts of brown seaweeds, also displays strong anti-inflammatory activity through their antioxidant activities (22, 23). However, there have been no studies focusing on the effects of low-molecular-weight fucoidan and fucoxanthin on intestinal inflammation. Therefore, we investigated the effects of low-molecular-weight fucoidan (LMF) and high-stability fucoxanthin (HS-Fucox) in a lipopolysaccharide (LPS)-induced inflammatory Caco-2 cell model adhering with *B. lactis*, which was undertaken to study the physiological barrier function and pharmacological cytokines secretion of LMF and/or HS-Fucox under intestinal inflammation.

## Materials and methods

### Materials

LMF (Hi-Q Oligo-Fucoidans^®^) and HS-Fucox were derived from *Sargassum hemiphyllum* and prepared by Hi-Q Marine Biotech International Ltd. (New Taipei City, Taiwan). LMF was obtained by enzyme hydrolysis of original fucoidan. The characteristics of LMF-LJ were average molecular weight of 0.8 KDa (92.1%), fucose content 210.9±3.3 µmol/g, and sulfate content 38.9±0.4% (w/w). The extraction method followed the method mentioned before with technological modifications (24). HS-Fucox is a mixture of brown seaweed extract containing about 10% of fucoxanthin that is coated directly with polysaccharides of its own. It was dissolved in double-distilled H_2_O (ddH_2_O) and completely dissolved by stirring at room temperature for 30 min.

### *Bifidobacterium lactis* cultivation and growth curve

*Bifidobacterium lactis BCRC* 17394 was purchased from the Bioresource Collection and Research Center, Hsinchu, Taiwan. This strain was subcultured in Man Rogosa and Sharpe medium (Scharlau) at 37°C under anaerobic conditions for 24 h, diluted with sterile saline to have the cell density of 1×10^8^ cfu/mL, and used as inoculums for the following cultivation experiments. *B. lactis* had an initial cell count of 4.5 log cfu/ml and cultured with various concentrations (0, 10, 50, 100, and 200 µg/ml) of LMF, HS-Fucox, and LMF+HS-Fucox for 48 h. LMF+HS-Fucox was a mixture with 50% LMF and 50% HS-Fucox. The cell counts of the sample groups were compared with the initial cell counts. The cell count measurement was carried by a serial 10-fold dilution, followed by spreading 0.1 ml of the diluents onto plate count agar plates. The plates were incubated at 37°C for 24 h prior to counting the colony to obtain cfu/ml.

### Cell line and culture condition

Caco-2 cell, the human intestinal epithelial cell, was obtained from the American Type Culture Collection. Caco-2 cells were cultured in Dulbecco's Modified Eagle's medium (DMEM), supplemented with a 15% fetal bovine serum (FBS) and 1% penicillin–streptomycin at 37°C in an atmosphere of 5% CO_2_. The medium was renewed every two days.

### Co-culture of Caco-2 cells and *B. lactis*

Caco-2 cells were grown for 12 days in six-well tissue culture plates to allow full differentiation to occur. At 12 h, prior to the addition of the *B. lactis*, the medium was aspirated and replaced with antibiotic-free DMEM. Caco-2 cells cultured in six-well plates were previously determined to contain 2×10^6^ cells/well, and 1 µg/ml LPS was added to each group, except the control group. 100 µl of *B. lactis* (10^6^ cfu/ml) was added with or without a sample onto Caco-2 cells in separate wells. The plates were incubated for 8 h at 37°C in an atmosphere of 5% CO_2_. After being incubated, transepithelial resistance (TER) was determined directly by meter, the supernatants were collected for cytokine secretion assay, and the Caco-2 cells were washed twice with 1-ml phosphate buffered saline (PBS), then for tight junction protein mRNA expression assay.

### Cell viability

The cell viability of cells was determined by 3-(4,5-Dimethylthiazol-2-Yl)-2,5-Diphenyltetrazolium Bromide (MTT) colorimetric assay (25). Cells were reacted with MTT (1 mg/ml) for 4 h, and absorbance was recorded at 570 nm (26). The cell viability (%) was determined as (A1/A0)×100%, where A0 and A1 were the absorbance of the control group (meaning, in the absence of sample and LPS treatment) and the sample group (0, 50, and 200 µg/ml with LPS treatment), respectively.

### TER assay

TER was measured in Ω cm^2^ using a Millicell ERS-2 Epithelial Volt-Ohm Meter (Millipore, Bedford, MA), by placing separate electrodes in the upper and lower wells according to the manufacturer's instructions (27). Monolayers showing TER values of 200–300 Ω cm^2^ were used for the experiments.

### Tight junction protein mRNA expression assay

Total RNA was isolated by RNAzol B (Amersham Pharmacia Biotech, Sweden), and the concentration of total RNA was detected by spectrophotometer (Hitachi, Japan). The synthesis of cDNA was performed using Improm-II TM Reverse Transcriptase (Promaga, WI, USA) according to the manufacturer's instructions. Polymerase chain reaction (PCR) was performed on the reverse-transcribed cDNA product to determine the expression of occludin, claudin-1, claudin-2, and β-actin (as an internal control) using a thermal cycler (Biometra, UNO-Thermoboblock, UK). The initial 1 min of 95°C denaturation was followed by the amplification sequence protocol of occludin (1 min of 55°C annealing and 3 min of 72°C extension), claudin-1 (1 min of 60°C annealing and 1 min of 72°C extension), claudin-2 (1 min of 56°C annealing and 1 min of 72°C extension), and β-actin for 30 cycles. Primers were listed 5′-3′ as follows: Occludin: F, TCA GGG AAT ATC CAC CTA TCA CTT CAG; R, CAT CAG CAG CAG CCA TGT ACT CTT CAC. Claudin-1: F, GCG CGA TAT TTC TTC TTG CAG G; R, TTC GTA CCT GGC ATT GAC TGG. Claudin-2: F, CTC CCT GGC CTG CAT TAT CTC; R, ACC TGC TAC CGC CAC TCT GT. β-actin: F, GAC TAC CTC ATG AAG ATC CT; R, CCA CAT CTG CTG GAA GGT GG (F: forward primer for sequence, R: reverse primer for sequence). The above primers were purchased from Mission Biotech Co., Ltd. (Taipei, Taiwan). The PCR products were separated by electrophoresis on 1.2% agarose gels and visualized by ethidium bromide staining under UV irradiation. The image of the resulting gel was captured and analyzed by ImageMaster VDS and ImageMaster 1D Elite software (Amersham Pharmacia Biotech, Sweden).

### Cytokine secretion assay

After cultivation of the Caco-2 cells and *B. lactis* as mentioned above, the supernatants were collected for IL-1β, IL-10, TNF-α, and IFN-γ. The concentrations of IL-1β, IL-10, TNF-α, and IFN-γ were determined by ELISA kits (R&D Systems Inc., Minneapolis, MN, USA) according to the manufacturer's instructions.

### Adhesion of *B. lactis* to Caco-2 cells by direct counting

The light microscopic observation of *B. lactis* was carried out by fixing washed cells in 100% methanol for 30 min followed by Gram staining. *B. lactis* is a Gram-positive cell that remains purple in color.

### Statistical assay

Numerical data are presented as means±standard deviation. The data were analyzed by a one-way analysis of variance (ANOVA) and followed by the least significant difference test using SPSS ver.10 (Chicago, IL) software.

## Result and discussion

### LMF-, HS-Fucox-, and LMF+HS-Fucox-activated probiotic growth

**Fig. 1:**
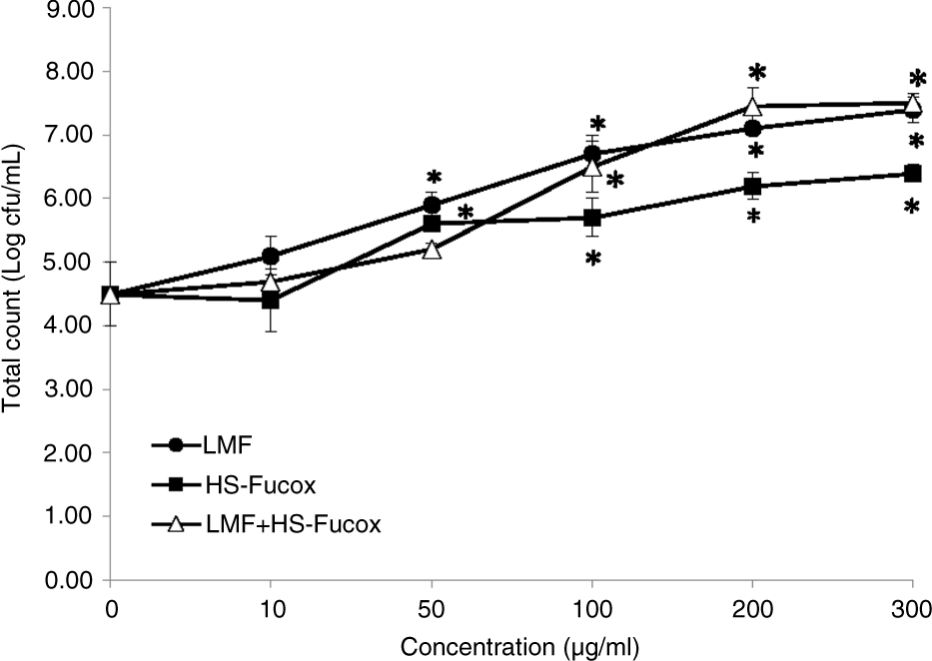
Effects of LMF, HS-Fucox, and LMF+HS-Fucox (0, 10, 50, 100, 200, and 300 µg/ml) on the growth of *B. lactis* at 37°C for 24 h. Values were expressed as mean±SD, *n*=5. *p<0.05 when compared with initial cell count.

Reports have revealed that *B. lactis*, as a probiotic, is protective against immune and infectious diseases (28, 29). It is used in probiotic food, particularly fermented milk products intended for medicinal use. Intestinal dysbiosis has increasingly been observed in a variety of intestinal and systemic diseases, and maintaining an adequate bacteria profile may be a key point for healthy intestinal barrier function. Prebiotics are used to selectively stimulate the growth of bifidobacteria and increase the body's natural resistance to invading pathogens (30). Hence, the effects of LMF, HS-Fucox, and LMF+HS-Fucox on *B. lactis* were examined. Our result revealed that LMF and LMF+HS-Fucox significantly promoted the growth of *B. lactis* at 50 µg/ml, whereas HS-Fucox showed significant promotion at 100 µg/ml. LMF and LMF+HS-Fucox had a better effect on the growth of *B. lactis* than HS-Fucox (Fig. 1). When the Caco-2 cells were cultured alone, their MTT absorbance value was 1.39±0.03. When the Caco-2 cells were co-cultured with *B. lactis* bacteria, the MTT absorbance value was 1.36±0.05. There were no significant differences between the two methods, and the co-culture showed no significant effect on cell growth (Fig. 1). Most prebiotics are water soluble or highly polar to stimulate the growth of probiotics, such as fucoidan (31) and laminarin derived from *Laminaria digitate* (32). Some reports demonstrated that oligosaccharides are the most appropriate prebiotics to be used in effective synbiotic formulations (33, 34). LMF, an oligosaccharide hydrolyzed from original fucoidan, appeared as a prebiotic to *B. lactis*. However, HS-Fucox stimulated the growth of *B. lactis* slightly, possibly because that phenolic compound was probably unsuitable as a prebiotic (35), and its prebiotic activity might come from the polysaccharide coating.

### LMF, HS-Fucox, and LMF+HS-Fucox against LPS-induced intestinal epithelial cell damage

**Fig. 2:**
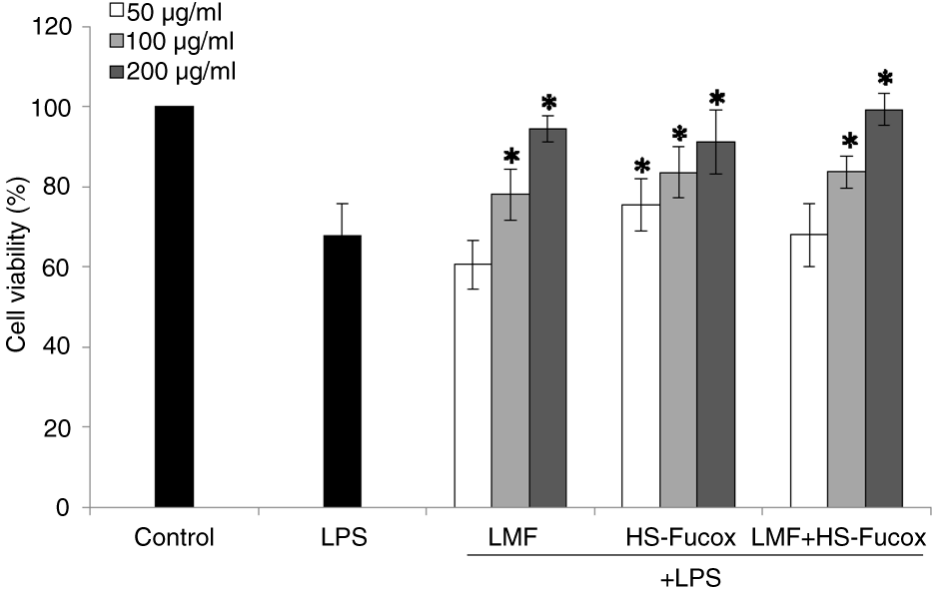
Effects of LMF, HS-Fucox, and LMF+HS-Fucox (50, 100, and 200 µg/ml) on the cell viability in the Caco-2 cells for 8 h, induced by LPS. The values were expressed as mean±SD, *n*=5. *p<0.05 when compared with LPS group alone.

**Fig. 3:**
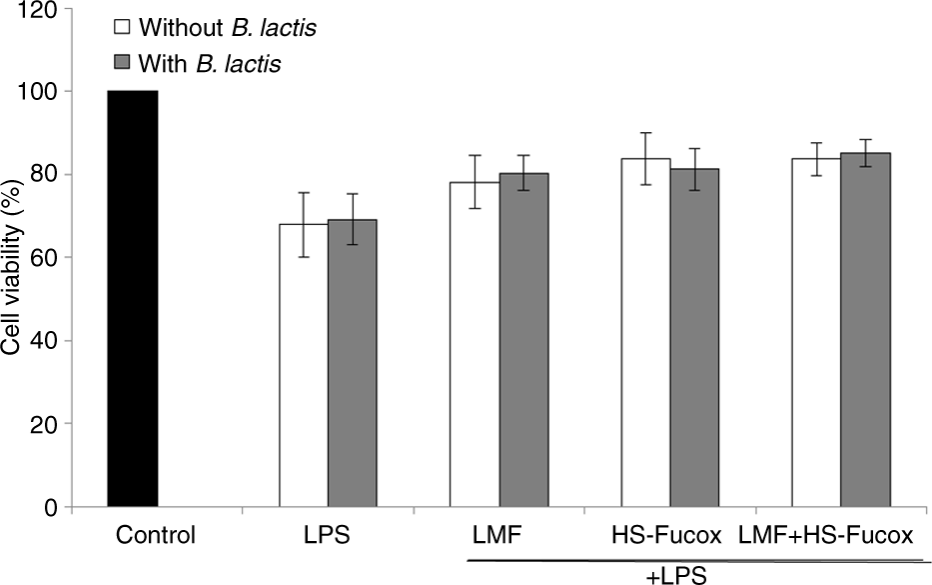
Effects of 100 µg/ml LMF, HS-Fucox, and LMF+HS-Fucox on cell viability in the co-culture of Caco-2 cells and *B. lactis* for 8 h, induced by LPS. The values were expressed as mean±SD, *n*=5. There was no significant difference with and without LPS in the *B. lactis* group.

LPS is produced by Gram-negative bacteria and can induce innate immune responses. The intestinal inflammation may serve as a nidus that can cause local and systemic organ dysfunction. In addition, LPS induced epithelial cell damage and mucosal hyperpermeability in vitro (36). First, we determined the effect of LMF, HS-Fucox, and LMF+HS-Fucox on the protective functions of Caco-2 cells. The percentage of cell viability decreased to 67.91% of the control medium in 1 µg/ml LPS. HS-Fucox significantly inhibited the LPS-induced cell damage at concentrations as low as 50 µg/ml, and LMF and LMF+HS-Fucox significantly inhibited the LPS-induced cell damage at a higher concentration of 100 µg/ml (Fig. 2). Sachindra et al. reported that the hydroxyl radical-scavenging activity of fucoxanthin was 13.5 times higher than that of α-tocopherol and showed the superiority of anti-inflammatory activity, such that HS-Fucox could inhibit the LPS-induced Caco-2 cell damage at a low concentration (37). Moreover, we were the first to use fucoxanthin on an intestinal inflammatory model. We also observed that LMF+HS-Fucox (99.34±3.94%) showed a stronger effect on increasing cell viability than LMF (94.56±3.26%) and HS-Fucox (91.33±7.99%) at 100 µg/ml, a result that matched our previous studies showing that HS-Fucox may efficiently scavenge reactive-oxygen species (ROS) (38) and that LMF reduced inflammation through the inhibition of NF-κB (39). Under the co-culture of Caco-2 cells and samples, cell viability showed no significant effect with or without *B. lactis* (Fig. 3), suggesting that *B. lactis* maintained the homeostasis within the dynamic ecosystem in the human body and had no direct effect on the intestinal cells. Therefore, the new natural bioactive compounds that enhance the probiotic properties and intestinal barrier functions are of prime importance.

### LMF, HS-Fucox, and LMF+HS-Fucox against LPS-induced destruction of intestinal epithelial barrier function

**Fig. 4:**
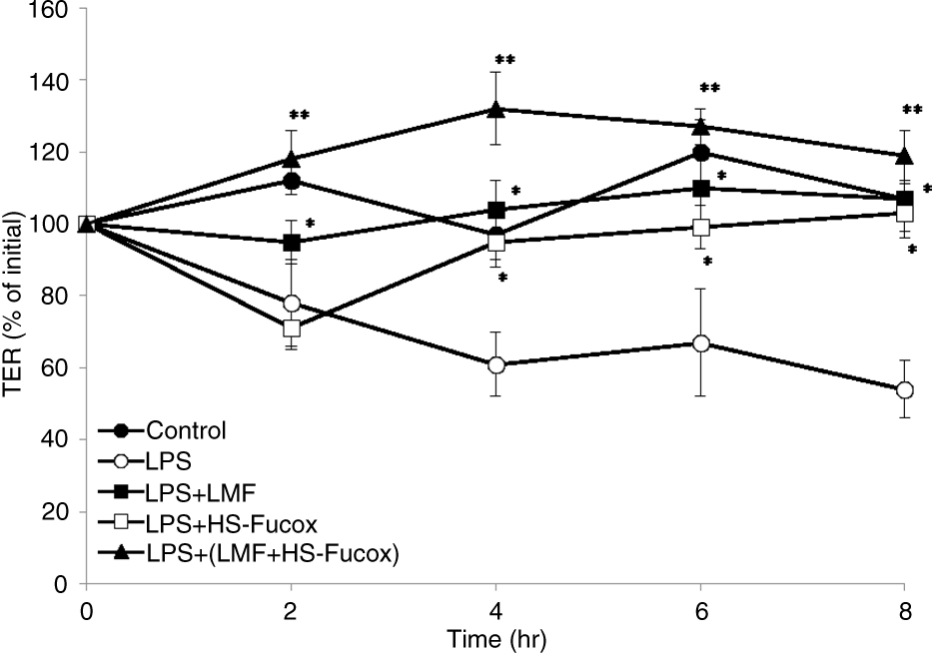
Effects of 100 µg/ml LMF, HS-Fucox, and LMF+HS-Fucox on the intestinal epithelial barrier function in the co-culture of Caco-2 cells and *B. lactis* for 8 h, induced by LPS. The intestinal epithelial barrier function was measured by TER assay. The values were expressed as mean±SD, *n*=5. *p<0.05, **p<0.01 when compared with LPS group alone.

The intestinal barrier occurs coincident with increasing the enteral feeding and establishing normal intestinal bacterial colonization. The mechanisms involved in the development of intestinal barrier function are probably multifactorial. Under the co-culture of the Caco-2 cells and *B. lactis* LPS-induced inflammatory system, we first determined the effect of samples on the protective functions of Caco-2 cell monolayers, and the integrity of polarized Caco-2 cell monolayers was determined by measuring the TER, which reflects the tightness of the tight junction between the epithelial cells (40). Through TER assay, it was shown that LPS destroyed the functions of Caco-2 cell monolayers, and the TER (percent of initial) was lower than that of the control group. However, when treating with 100 µg/ml LMF, HS-Fucox, and LMF+HS-Fucox, the TER continued to increase for an additional 8 h and remained constant from 4 to 8 h. Among them, LMF+HS-Fucox significantly increased the TER (**p<0.01 when compared with LPS group alone) and was followed by LMF and HS-Fucox (*p<0.05 when compared with LPS group alone), indicating an enhancement of the intestinal epithelial barrier function (Fig. 4).

Occludin is a 65-kDa integral plasma-membrane protein and, as the main component of tight junctions, maintains the morphological stability in epithelial tissues (41). Claudin-1 and claudin-2 are smaller (20–27 kDa) transmembrane proteins, and occludin constitutes tight junction strands as multiple integral membrane proteins with four putative transmembrane domains (42). Because the tight junctions act as an intercellular gate, we next focused on the tight junction proteins, occludin, claudin-1, and claudin-2 mRNA expression under LPS-induced inflammation.

**Fig. 5:**
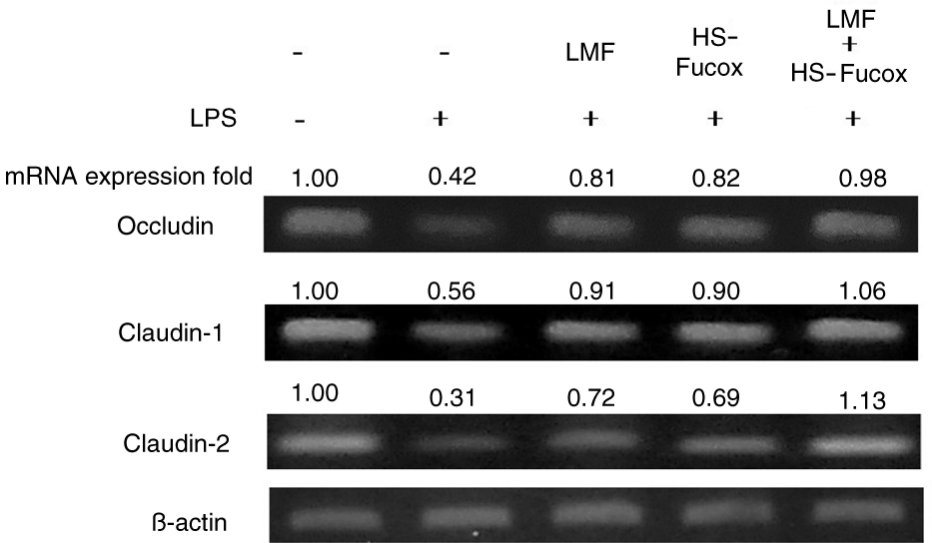
Effects of 100 µg/ml LMF, HS-Fucox, and LMF+HS-Fucox on occludin, claudin-1, and claudin-2 mRNA expression and its relative fold in the co-culture of Caco-2 cells and *B. lactis* for 8 h, induced by LPS. Values were expressed as mean±SD, *n*=5.

As expected, LPS inhibited occludin, claudin-1, and claudin-2 mRNA expression. After treating with 100 µg/ml LMF, HS-Fucox, and LMF+HS-Fucox, the mRNA expressions were recovered (Fig. 5). Iraha et al. also demonstrated that fucoidan can significantly increase the expression of claudin-1 and claudin-2 under H_2_O_2_ disrupting epithelial barrier function (40). In addition to fucoidan, flavonoid from plant components can enhance barrier function by upregulating claudin-4 expression (43). Moreover, our data showed that the occludin, claudin-1, and claudin-2 expressions were significant greater in the LMF+HS-Fucox (**p<0.01) group than in the LMF (*p<0.05) and HS-Fucox (*p<0.05) groups. This result matched the consequence of Fig. 4. So, it was suggested that LMF+HS-Fucox directly induced the expression of some tight junction proteins and might contribute to the enhancement of the epithelial barrier function. For comparison, by adding LMF with LPS treatment, the increasing trend of TER and tight junction protein expressions was only slightly higher than that of the HS-Fucox group. This result indicated that LMF and HS-Fucox exhibited a protective effect on epithelial cell injury, and HS-Fucox offered scavenging ROS activity (38) strong enough to make LMF+HS-Fucox an appropriate therapy for the treatment of inflammatory bowel diseases.

### LMF, HS-Fucox, and LMF+HS-Fucox modulated LPS-induced immune disorder

**Fig. 6:**
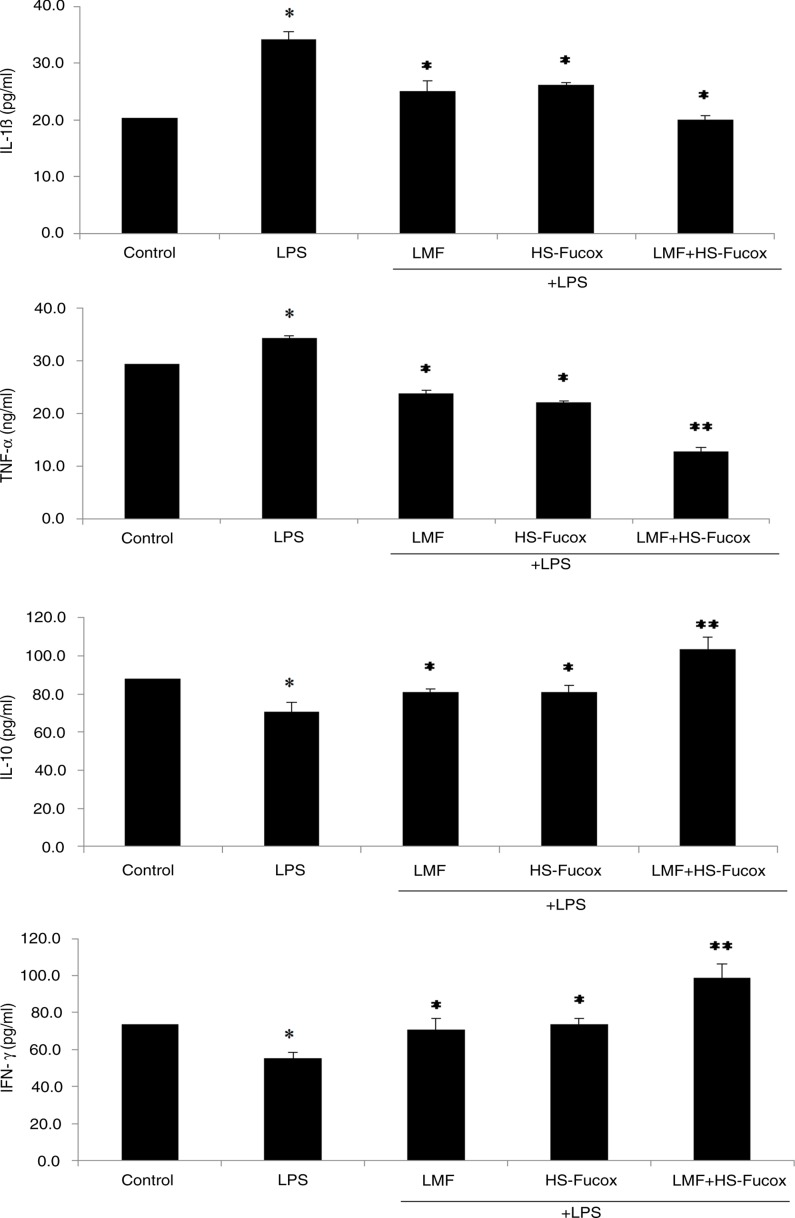
Effects of 100 µg/ml LMF, HS-Fucox, and LMF+HS-Fucox on IL-1β, IL-10, TNF-α, and IFN-γ production in the co-culture of Caco-2 cells and *B. lactis* for 8 h, induced by LPS. Values were expressed as mean±SD, *n*=5. *p<0.05, **p<0.01 when compared with the LPS group alone.

The pro-inflammatory cytokines secreted by the epithelium, such as IL-1β and TNF-α, are hallmarks of inflammatory responses in the intestine. Relatively, the intestine also secrets anti-inflammatory cytokines, namely IL-10 and IFN-γ, that regulate cell inflammation (44). As shown in Fig. 6, LMF, HS-Fucox, and LMF+HS-Fucox were potent inhibitors of IL-1β and TNF-α and promoters of IL-10 and IFN-γ. It seems that LMF, HS-Fucox, and LMF+HS-Fucox were trying to balance the immune disorder under LPS-induced inflammation. To examine the main contributors to regulate the inflammation, LMF, HS-Fucox, and LMF+HS-Fucox were separately used. LMF (*p<0.05) and HS-Fucox (*p<0.05) exhibited similar effects on IL-1β, TNF-α, IL-10, and IFN-γ. However, when LMF and HS-Fucox (LMF+HS-Fucox group, **p<0.01) are combined together, the anti-inflammatory activity became greater. Fucoidan and fucoxanthin both appeared to reduce the level of pro-inflammatory mediators, including IL-1β and TNF-α via the inhibition of NF-κB activation (39, 45). We suggested that LMF+HS-Fucox may provide comprehensive inhibition of the expressions of inflammatory cytokines by NF-κB pathway and probably possess anti-inflammatory properties for other immunity, via similar pathways in the epithelial cell (46). However, the mechanism still needs further study.

### LMF, HS-Fucox, and LMF+HS-Fucox enhanced *B. lactis* adhesion to intestinal epithelial cells

**Fig. 7:**
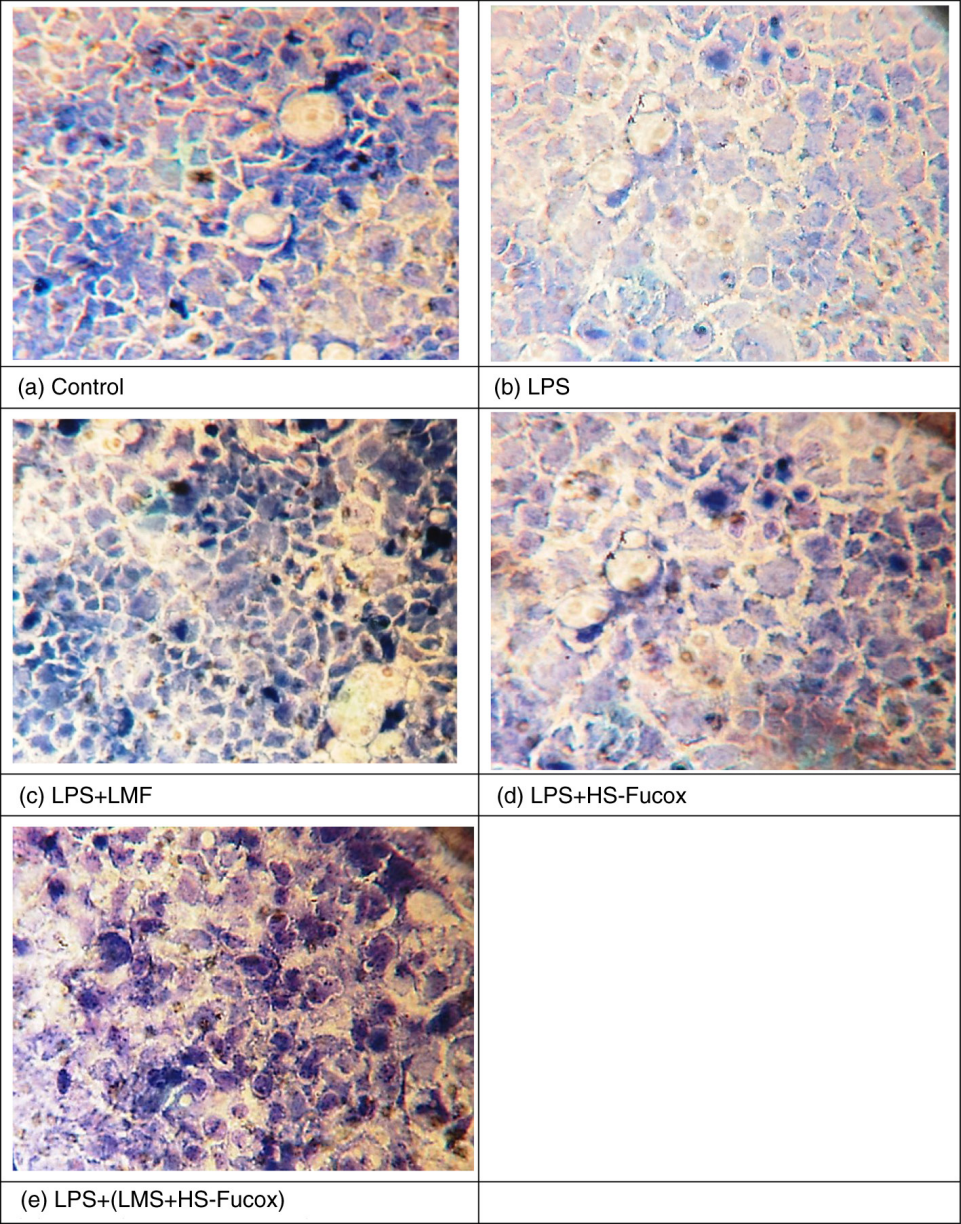
Gram staining for 100 µg/ml LMF, HS-Fucox, and LMF+HS-Fucox in the co-culture of Caco-2 cells and *B. lactis* for 8 h, induced by LPS. *B. lactis* is a Gram-positive cell that remains purple in color. (a) Control, (b) LPS, (c) LPS+LMF, (d) LPS+HS-Fucox, and (e) LPS+(LMF+HS-Fucox).

Gram staining is a method of staining used to differentiate bacterial species into two large groups, Gram positive and Gram negative, and *B. lactis*, a kind of Gram-positive cell, appear in purple color. Observation of the Gram-stained cells under a light microscope showed that LPS treatment reduced the *B. lactis* counts of the Caco-2 cells the co-culture system, which appeared with a very slight blue/purple color (Fig. 7b). In contrast, a clear purple color appeared when treating with LMF, HS-Fucox, and LMF+HS-Fucox (Fig. 7c, d, and e). The LMF-treated group was inducing more *B. lactis* adhered to Caco-2 cells than the HS-Fucox-treated group (Fig. 7c and d). This result is in agreement with the findings of Fig. 1, in which LMF appeared as a prebiotic to *B. lactis*, and HS-Fucox was probably unsuitable as prebiotic. Furthermore, brown seaweed extracts have been proved to reduce the enterobacteriaceae populations and enhance the IL-6 and IL-8 cytokine expression to an ex vivo LPS challenge (32), and improve the probiotic properties of *Lactobacillus plantarum* (47). The LMF+HS-Fucox-treated group showed stronger activity of *B. lactis* adhesion than the results in the LMF- and HS-Fucox-treated groups, suggesting that LMF+HS-Fucox was important to the *B. lactis* adhesion to Caco-2 cells, not only for their prebiotic effect but also because of many of their functional properties.

In conclusion, LMF and HS-Fucox were proved to enhance the functions of the immune system by inhibiting IL-1β and TNF-α and promoting IL-10 and IFN-γ, and revealed an anti-inflammatory effect in the intestinal cell line. The present findings suggested that LMF and HS-Fucox alone or in combination could be used as potential therapeutic agents in the treatment of intestinal inflammation.
